# Antimicrobial Applications of Transition Metal Complexes of Benzothiazole Based Terpolymer: Synthesis, Characterization, and Effect on Bacterial and Fungal Strains

**DOI:** 10.1155/2014/764085

**Published:** 2014-09-14

**Authors:** Mohamed A. Riswan Ahamed, Raja S. Azarudeen, N. Mujafar Kani

**Affiliations:** ^1^Department of Chemistry, Oxford Engineering College, Tiruchirappalli, Tamil Nadu 620 009, India; ^2^Department of Chemistry, Coimbatore Institute of Technology, Coimbatore, Tamil Nadu 641 014, India; ^3^PG and Research Department of Chemistry, Jamal Mohamed College (Autonomous), Tiruchirappalli, Tamil Nadu 620 020, India

## Abstract

Terpolymer of 2-amino-6-nitro-benzothiazole-ethylenediamine-formaldehyde (BEF) has been synthesized and characterized by elemental analysis and various spectral techniques like FTIR, UV-Visible, and ^1^H and ^13^C-NMR. The terpolymer metal complexes were prepared with Cu^2+^, Ni^2+^, and Zn^2+^ metal ions using BEF terpolymer as a ligand. The complexes have been characterized by elemental analysis and IR, UV-Visible, ESR, ^1^H-NMR, and ^13^C-NMR spectral studies. Gel permeation chromatography was used to determine the molecular weight of the ligand. The surface features and crystalline behavior of the ligand and its complexes were analyzed by scanning electron microscope and X-ray diffraction methods. Thermogravimetric analysis was used to analyze the thermal stability of the ligand and its metal complexes. Kinetic parameters such as activation energy (*E*_*a*_) and order of reaction (*n*) and thermodynamic parameters, namely, Δ*S*, Δ*F*, S*, and *Z*, were calculated using Freeman-Carroll (FC), Sharp-Wentworth (SW), and Phadnis-Deshpande (PD) methods. Thermal degradation model of the terpolymer and its metal complexes was also proposed using PD method. Biological activities of the ligand and its complexes were tested against *Shigella sonnei*, *Escherichia coli*, *Klebsiella* species, *Staphylococcus aureus*, *Bacillus subtilis*, and *Salmonella typhimurium* bacteria and *Aspergillus flavus*, *Aspergillus niger*, *Penicillium* species, *Candida albicans*, *Cryptococcus neoformans*, *Mucor species* fungi.

## 1. Introduction

The construction of polymers with d-block transition metal complexes has been developed rapidly in recent years, owing to their interesting high thermal stability and enormous pharmacological activity along with potential applications as functional materials. Owing to the high thermal stability of the polymers, they have adverse applications such as in waste water treatment for metal recovery, protective coatings, thermally stable materials, water disinfectants, antifouling paints, antimicrobial and surgical materials, gels and ointments for medical uses, and biological activity [1, 2].

Based on the survey made in the literature, it has been found that good work has been reported on polymers/copolymers/terpolymers acting as various types of ligands with transition metal ions [3–5]. The chemical properties of benzothiazole based compounds have been intensively investigated in several research fields especially in polymer field, because of their high thermal stability and pharmacological activity [6, 7]. The thiazole ring dramatically increases the diversity of certain biological properties such as antibacterial [8], antiviral [9], and antitubercular [10] activities. These activities are probably due to the presence of the –N=C–S group present in the thiazole moiety [11].

A series of 4-isopropylthiazole-2-carbohydrazide analogs, derived from clubbed oxadiazole-thiazole and triazole-thiazole derivatives, have been synthesized and characterized by Mallikarjuna et al. [12]. The synthesized compounds were evaluated for their preliminary* in vitro* antibacterial, antifungal, and antitubercular activity against* Mycobacterium tuberculosis* H_37_Rv strain by broth dilution assay method. Ahamad et al. [13] reported a new class of metal chelated polyurea for its excellent antimicrobial activity against* S. aureus*,* E. coli*,* B. subtilis*, and* S. typhi*. The Cu^2+^ chelated polyurea shows higher zone of inhibition than the other ones due to higher stability constant and may be used in biomedical applications. Patel et al. [14] reported that the synthesis of low molecular weight polymeric resins contains several functional groups in gaining importance to involve the antimicrobial activity. All the synthesized polymers were examined for the antimicrobial activity against bacteria (*Escherichia coli*,* Bacillus subtilis*, and* Staphylococcus citreus*), fungi (*Aspergillus niger*,* Sporotrichum pulverulentum*, and* Trichoderma lignorum*), and yeast (*Candida utilis*,* Saccharomyces cerevisiae*, and* Pichia stipitis*). It was observed that most of the polymers could be used as antibacterial and antifungal agents. Alagawadi and Alegaon [15] synthesized series of novel 5-substituted-2,4-thiazolidinedione derivatives and studied the antimicrobial screening tests which reveal the new compounds that act as antibacterial and antifungal agents. Ashok et al. [16] accounted for the one pot synthesis of some novel derivatives of thiazolo[2,3-b]dihydropyrimidinone possessing 4-methylthiophenyl moiety and evaluation of their antibacterial and antifungal activities. They have exhibited moderate to excellent growth inhibition against the chosen bacteria and fungi due to the presence of N and S donor atoms present in the antimicrobial agents. Ahamad et al. [17] studied the* in vitro *antibacterial and antifungal assay of poly-(ethylene oxamide-*N*,*N*′-diacetate) and its polymer-metal complexes. The polymer-metal complexes showed excellent antibacterial activities against both types of microorganisms; the polymeric ligand was also found to be effective but less so than the polymer-metal complexes. On the basis of the antimicrobial behavior, these polymers may be used as antifungal and antifouling coating materials in fields like life-saving medical devices and the bottoms of ships. Recently, our research group synthesized new types of polymer and oligomer ligands and their transition metal complexes for their excellent thermal stability and antimicrobial activity [18–20].

The present paper aims to introduce the new 2-amino-6-nitro-benzothiazole-ethylenediamine-formaldehyde (BEF) terpolymer ligand and its complexes. It provides the synthesis, characterization, thermal properties, and biological activities of the 2-amino-6-nitro-benzothiazole-ethylenediamine-formaldehyde (BEF) terpolymer ligand and its complexes. The physicochemical properties and characterization of the BEF terpolymer ligand and its complexes were carried out by elemental analysis, molar conductivity, magnetic susceptibilities, GPC, FTIR, UV-Visible, ESR, ^1^H and ^13^C NMR, SEM, XRD, and TGA. From the TGA data, the various kinetic and thermodynamic parameters were evaluated for the BEF terpolymer ligand and its complexes. The decomposition model for the thermal degradation reaction was also proposed. The antimicrobial activities of the terpolymer ligand and its complexes were reported against six bacterial strains such as* Shigella sonnei*,* Escherichia coli*,* Klebsiella* species (NCIM 2719),* Staphylococcus aureus* (ATCC 25923),* Bacillus subtilis* (ATCC 6633), and* Salmonella typhimurium* (ATCC 23564) and six fungal strains such as* Aspergillus flavus*,* Aspergillus niger*,* Penicillium *species,* Candida albicans*,* Cryptococcus neoformans*, and* Mucor* species.

## 2. Experimental

### 2.1. Materials

2-Amino-6-nitro-benzothiazole and ethylenediamine were procured from Sigma Aldrich, USA, and used as received. Formaldehyde (37%) was procured from Merck, India, and used as received. Double distilled water was used for all the experiments. All other chemicals and metal salts were of analytical grade and were used without further purification.

### 2.2. Synthesis of Terpolymer Ligand

**Scheme 1:**
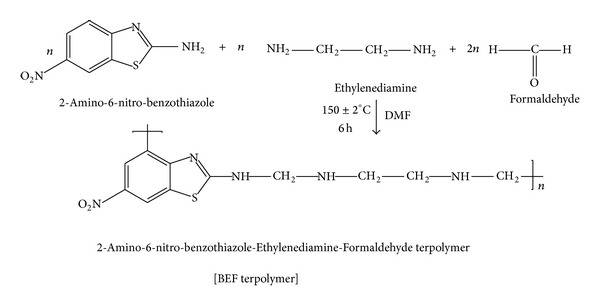
Reaction route of the BEF terpolymer ligand.

A collective mixture of 2-amino-6-nitro-benzothiazole (9.76 g, 0.05 M) and ethylenediamine (8.12 mL, 0.05 M) with formaldehyde (8.11 mL, 0.1 M) was taken as monomers in a clean round bottom flask equipped with a mechanical stirrer and a refluxed condenser using dimethyl formamide (DMF) as a reaction medium in 1 : 1 : 2 mole ratio. The homogeneous mixture was refluxed in an oil bath at 150 ± 2°C with constant stirring for 6 h. After the reaction time was over, the resultant mixture was cooled and then poured into crushed ice with vigorous shaking. The obtained precipitate was separated out by filtration, washed with hot water, ether, and methanol to remove the unreacted monomers, and recrystallized from tetrahydrofuran (THF). The route of synthesis for the BEF terpolymer is shown in Scheme 1.

### 2.3. Preparation of Metal Complexes

**Scheme 2:**
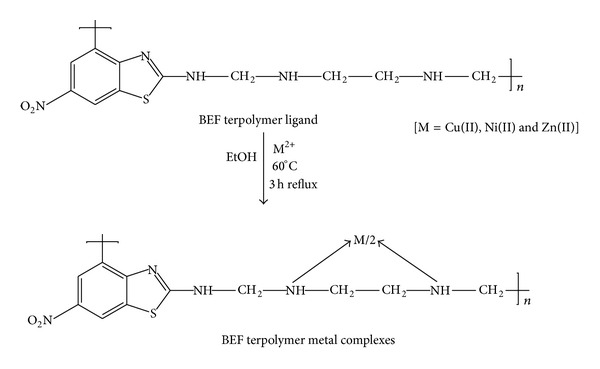
Reaction route of the BEF metal complexes.

The terpolymer metal complexes have been prepared using the synthesized terpolymers as ligand with few transition metal ions such as Cu^2+^, Ni^2+^, and Zn^2+^ ions. The BEF terpolymer (2 g) was taken in a round bottom flask and immersed for 2 h in ethanol solution for swelling. The cupric nitrate (1 g) was dissolved in ethanol solution and then poured into round bottom flask equipped with mechanical stirrer and a reflux condenser. The reaction mixture was refluxed at 60°C for 3 h. The obtained colloidal precipitate in the flask was separated out. The product was then filtered off and washed with ether and ethanol to remove the impurities. This process has been repeated several times to separate the purified product. The resultant purified sample was air dried, powdered, and kept in vacuum desiccator with silica gel. The same procedure was also followed for the preparation of BEF complexes with Ni^2+^ and Zn^2+^ metal ions in the form of their nitrate salts. The scheme of preparation of the BEF complex with Cu^2+^, Ni^2+^, and Zn^2+^ metal ions is shown in Scheme 2.

### 2.4. Elemental Analysis

The elemental analysis has been carried out using Elementar instrument, model Vario EL III, Germany. The percentage of elements such as carbon (C), hydrogen (H), nitrogen (N), and sulphur (S) present in the 2-amino-6-nitro-benzothiazole-ethylenediamine-formaldehyde (BEF) terpolymer and its metal complexes with the metal ion content was determined.

### 2.5. Gel Permeation Chromatography

The number average $\left(\bar{M_{n}}\right)$, weight average $\left(\bar{M_{w}}\right)$, and size average $\left(\bar{M_{z}}\right)$ molecular weights of terpolymer were measured by gel permeation chromatography (Shimadzu, Japan) using tetrahydrofuran (THF) as a mobile phase and CLC polystyrene as a stationary phase in the column.

### 2.6. Spectral Analyses

The UV-Visible spectra of the terpolymers and its metal complexes were recorded in Shimadzu (model 1601PC) UV-Visible spectrophotometer, Japan. The ESR spectra of the terpolymer and its metal complexes were recorded in Bruker instrument, model EleXsys E 500, USA. The FTIR spectra of the terpolymer and its metal complexes were obtained with Avatar (model 330, USA) FTIR spectrometer using KBr. The ^1^H and ^13^C NMR spectra were recorded on a Bruker 400 MHz (USA) NMR spectrometer, using DMSO-d_6_ as a solvent.

### 2.7. SEM and XRD Methods

The surface analysis of the BEF terpolymer and its metal complexes was examined at different magnifications by scanning electron microscope using Hitachi instrument, model S-3000H, Japan. The XRD analysis of the terpolymer and its metal complexes was performed using PANalytical instrument (X'Pert PRO, The Netherlands).

### 2.8. Thermogravimetric Analysis

The thermal stability of the BEF terpolymer and its metal complexes was observed by thermogravimetric analysis using TA instruments, model SDT Q600, USA, at a heating rate of 20°C/min in a static nitrogen atmosphere.

#### 2.8.1. Thermal Degradation Kinetics and Mechanism

Sharp-Wentworth and Freeman-Carroll methods have been employed for the determination of kinetic and thermodynamic parameters such as order of reaction (*n*), activation energy (*E*_*a*_), entropy change (Δ*S*), free energy change (Δ*F*), apparent entropy (S*), and frequency factor (*Z*). Phadnis-Deshpande method has also been used to propose the thermal degradation mechanism for the synthesized terpolymer and its metal complexes [20].

### 2.9. Antimicrobial Analysis by Disc Diffusion Method

The BEF terpolymer and its metal complexes were screened for their antimicrobial activity by disc diffusion method [21] against six bacterial strains such as* Shigella sonnei*,* Escherichia coli*,* Klebsiella* species (NCIM 2719),* Staphylococcus aureus* (ATCC 25923),* Bacillus subtilis* (ATCC 6633), and* Salmonella typhimurium* (ATCC 23564) and six fungal strains such as* Aspergillus flavus*,* Aspergillus niger*,* Penicillium* species,* Candida albicans*,* Cryptococcus neoformans*, and* Mucor* species. Antimicrobial activity was tested by the filter paper disc diffusion technique involving the cultures of the selected organisms for 24 h. Mueller Hinton agar number 2 (Hi Media, India) was used as the bacteriological medium and sterile yeast nitrogen base with 2% agar (Hi Media, India) was used as the fungal medium. The test solutions of the terpolymer and its metal complexes were prepared in sterile dimethyl sulfoxide (DMSO) solvent for the study. The synthesized terpolymer and its metal complexes were tested at different concentrations ranging from 50 to 1000 ppm to find out the minimum concentration of the terpolymer ligand and its metal complexes required for inhibiting the growth of microbes.

Amoxicillin (100 *μ*g/mL) was taken as the standard for antibacterial activity. The organism was seeded into sterile nutrient agar medium by mixing one mL of inoculum with 20 mL sterile melted nutrient agar kept at 48–50°C in a sterile petri dish. The medium was allowed to solidify first. Then the test solutions, the standard drugs, and the blank were impregnated in whatman filter paper discs, placed on the solidified medium in the petri dish, and left undisturbed for 2 h at room temperature. The petri dishes were then incubated at 37°C for 24 h and the zone of inhibition for the test samples, standard, and control (DMSO) was measured.

Sterile yeast nitrogen base (Hi Media) with 2% agar was inoculated by a rotating swab (soaked in standard inoculum suspension) over the surface of the media. Fluconazole (100 mcg/mL) was taken as the standard for antifungal activity. The test solution impregnated discs were placed on the agar and were incubated at 37°C for 18 h. The zone of inhibition was measured by measuring the minimum dimension of the zone of fungal growth around the filter paper disc.

## 3. Results and Discussion

### 3.1. Physicochemical and Analytical Data

The physicochemical and analytical data of the BEF terpolymer and its metal complexes are given in Table 1. The BEF terpolymer is yellow in colour and its metal complexes are brown for Cu^2+^ complex and yellow for Ni^2+^ and Zn^2+^ complexes. The yield of the BEF terpolymer ligand was 79% and the yield of Cu^2+^, Ni^2+^, and Zn^2+^ complexes was 80%, 77%, and 78%, respectively. Based on the analytical data from the elemental analysis, the empirical formula of the BEF terpolymer ligand and its metal complexes is found to be in good agreement with the calculated elemental values of C, H, N, S, and M. The amount of metal ions present in the metal complexes indicates 1 : 2 M/L stoichiometry suggesting six coordination involving two coordinated water molecules for BEF–Cu, BEF–Ni, and BEF–Zn complexes. From the results, the general empirical formula of the repeating unit of the BEF terpolymer is C_11_H_13_N_5_O_2_S and of the metal complexes is C_22_H_26_N_10_O_4_S_2_Cu*·*2H_2_O for BEF–Cu, C_22_H_26_N_10_O_4_S_2_Ni*·*2H_2_O for BEF–Ni, and C_22_H_26_N_10_O_4_S_2_Zn*·*2H_2_O for BEF–Zn. From the molar conductance values, the metal complexes were found to be electrolytes.

### 3.2. Molecular Weight Measurements

The average molecular weight of the BEF terpolymer was determined by gel permeation chromatography. The weight average $\left(\bar{M_{w}}\right)$ and number average $\left(\bar{M_{n}}\right)$ molecular weight of the terpolymer were found to be 1941 and 1940, respectively. The polydispersity index $\left(\bar{M_{w}}/\bar{M_{n}}\right)$ was found to be 1.0005. The average molecular weight $\left(\bar{M_{z}}\right)$ of the terpolymer was found to be 1942. The polydispersity index $\left(\bar{M_{z}}/\bar{M_{w}}\right)$ of the terpolymer is 1.0005. The polydispersity index $\left(\bar{M_{w}}/\bar{M_{n}}\right)$ and $\left(\bar{M_{z}}/\bar{M_{w}}\right)$ values for the terpolymer indicate the narrow distribution of molecular weight in the terpolymer.

### 3.3. FTIR Spectra

**Figure 1:**
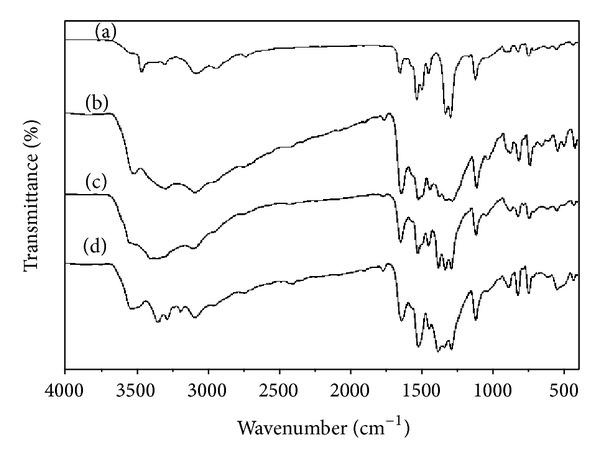
FTIR spectra of (a) BEF, (b) BEF–Cu, (c) BEF–Ni, and (d) BEF–Zn.

The FTIR spectra of BEF terpolymer ligand and its metal complexes are depicted in Figure 1 and the data are presented in Table 2. The band frequencies and the groups assigned to both terpolymer ligand and its metal complexes are based on the earlier literature [22, 23]. The ligand spectrum showed a band appearing in the range of 3455–3297 cm^−1^ is assigned to the –NH asymmetric and symmetric vibrations. The 2,6,8-trisubstituted benzothiazole ring is confirmed by sharp and medium absorption bands appearing between 1296 and 825 cm^−1^. A strong band that appeared at 1532 cm^−1^ is due to the –NH bending vibration. A band that appeared at 2731 cm^−1^ is assigned to the –CH stretching vibrations present in the aromatic ring. The bands appearing in the region of 3078–2936 cm^−1^ are attributed to –CH_2_ asymmetric and symmetric vibrations present in the terpolymer ligand. The presence of –CH_2_ bending vibration in N–CH_2_–CH_2_–N bridge in the spectrum is confirmed by the absorption band that appeared at 1450 cm^−1^.

In the spectra of BEF metal complexes, the bands are slightly broadened compared to the terpolymer ligand. The band that appeared at 1532 cm^−1^ is assigned to –NH bending vibrations in the ligand spectrum that is shifted to the lower frequencies (1521 to 1527 cm^−1^) in the case of polymer-metal complexes. This shift is due to the coordination of metal ions through the lone pair of both nitrogen atoms present in the ethylenediamine moiety. It is a clear evidence for the involvement of nitrogen atoms in the chelation. This is further supported by the appearance of *ν* N→M stretching vibration at 432–433 cm^−1^.

### 3.4. Electronic and ESR Spectra and Magnetic Moments

**Figure 2:**
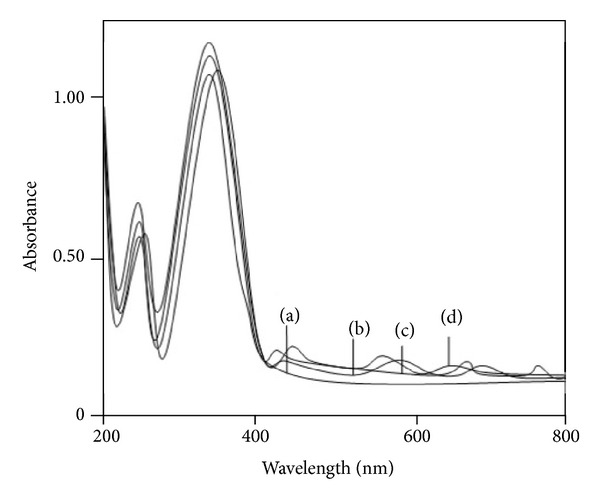
Electronic spectra of (a) BEF ligand, (b) BEF–Cu, (c) BEF–Ni, and (d) BEF–Zn.

The electronic spectra of the BEF terpolymer and its metal complexes are shown in Figure 2 and the data are presented in Table 3. The spectrum of the ligand showed two bands at 260 and 370 nm. These observed positions for the absorption bands have different intensities. The less intense band 260 nm is due to (*π* → π*) allowed transition of chromophore groups like NO_2_, >C=N, and C=C, which are in conjugation with an aromatic nucleus (benzothiazole ring), and the more intense band 370 nm may be due to (*n* → π*) transition of –NH groups. Thus *π* → π* transition indicates the presence of aromatic nucleus and *n* → π* transition indicates the presence of –NH groups. The electronic spectrum of BEF–Cu metal complex exhibited bands at 14295, 16465, and 24580 cm^−1^ and the assignments are ^1^ *B*_*g*_ → ^2^ *E*_*g*_, ^1^ *B*_*g*_ → ^2^ *E*_*g*_, and charge transfer spectra which lead to a distorted octahedral geometry with the magnetic moment of 1.85 B.M. The electronic spectrum of BEF–Ni exhibits three bands at 12474, 14642, and 24370 cm^−1^ assigned to the spin allowed transitions ^3^ *T*_2*g*_(*F*) ← ^3^ *A*_2*g*_(*F*), ^3^ *T*_1*g*_(*F*) ← ^3^ *A*_2*g*_(*F*), and ^3^ *T*_1*g*_(*P*) ← ^3^ *A*_2*g*_(*F*) in an octahedral environment with a magnetic moment of 3.05 B.M. The diamagnetic nature of the BEF–Zn is also confirmed with octahedral geometry. Based on the literature, the geometry is assigned to all the metal complexes [24].

**Figure 3:**
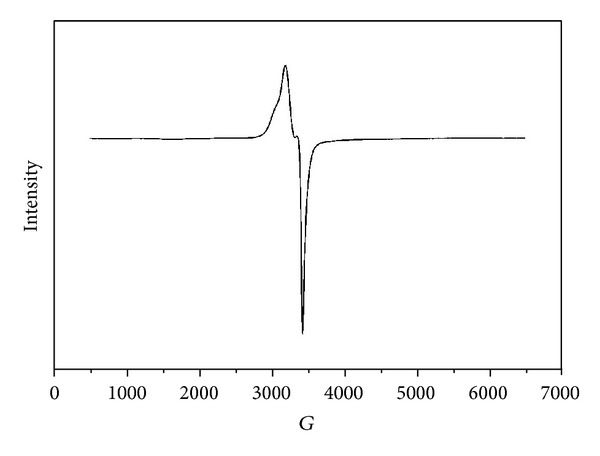
ESR spectra of BEF–Cu complex.

The ESR spectrum of BEF–Cu complex is shown in Figure 3 and its spectral data are presented in Table 3. The spectrum provides useful information about the metal ion environment in the respective complexes. The ESR spectrum of BEF–Cu metal complex showed that *g*_||_ = 2.21 and *g*_⊥_ = 2.11 lead to an octahedral environment. According to Kivelson and Neimen [25] the bond between the ligand and the metal possesses a more covalent character than ionic. In general, both the parallel and the perpendicular *g* values can be used to calculate the *G* value by the following equation:

(1) $$\begin{array}{c} G=\frac{g_{||}-2.002}{g_{⊥}-2.002}. \end{array}$$

Using the above equation, the *G* value for BEF–Cu was 1.92 which clearly indicates that the complex forms with a strong field ligand.

### 3.5. Nuclear Magnetic Resonance

The NMR (^1^H and ^13^C) spectra for BEF terpolymer ligand and its zinc metal complex (due to d^9^ and d^8^ configurations, both NMR spectra for copper and nickel metal complexes are complicated) were recorded to elucidate the structure of the ligand and its complex.

#### 3.5.1. ^1^H NMR Spectra

**Figure 4:**
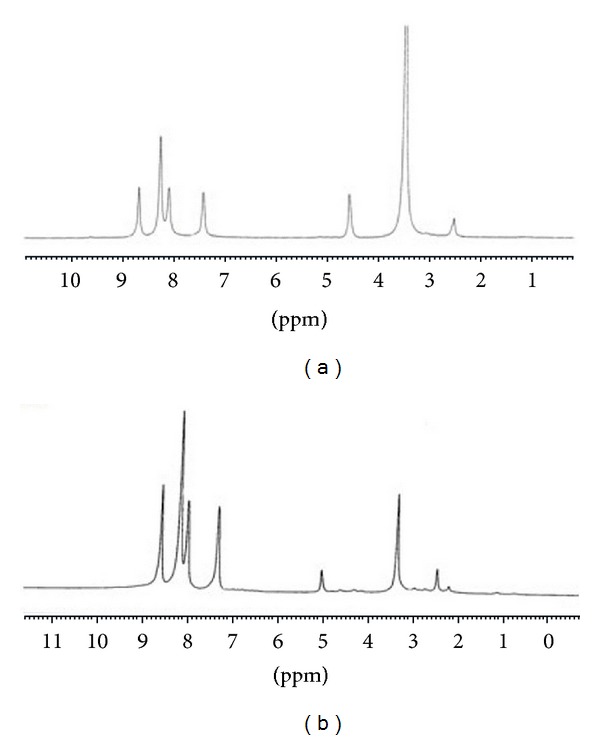
^1^H-NMR spectra of (a) BEF ligand and (b) BEF–Zn.

The ^1^H NMR spectra of BEF terpolymer ligand and its Zn^2+^ metal complex are shown in Figure 4. The signals obtained for the terpolymer and its metal complexes were interpreted on the basis of the data available in the literature [22, 26]. The spectrum of terpolymer showed that the signals appearing in the region of 7.39–8.24 ppm are assigned to all the protons of the aromatic ring. A peak appearing at 2.50 is assigned to the methylene protons of the terpolymer ligand. The terpolymer spectrum showed a signal appearing at 4.72 ppm that is assigned to the –NH protons of ethylenediamine moiety and the peak appearing at 8.65 ppm is assigned to –NH proton of thiazole ring. A weak signal appearing at 3.02 ppm may be assigned to ethylenic protons of an Ar–CH_2_–NH–CH_2_ moiety.

The spectrum of the Zn^2+^ complex showed multiplet in the range of 7.40–8.24 ppm is assigned to the aromatic protons and the methylene protons appeared at 2.15–2.50 ppm. The signal obtained at 5.08 ppm is assigned to –NH protons of ethylenediamine and this shift from its ligand gives a clear evidence for the complexation of the ligand with the metal ions through the lone pair of nitrogen of ethylenediamine moiety.

#### 3.5.2. ^13^C NMR Spectra

**Figure 5:**
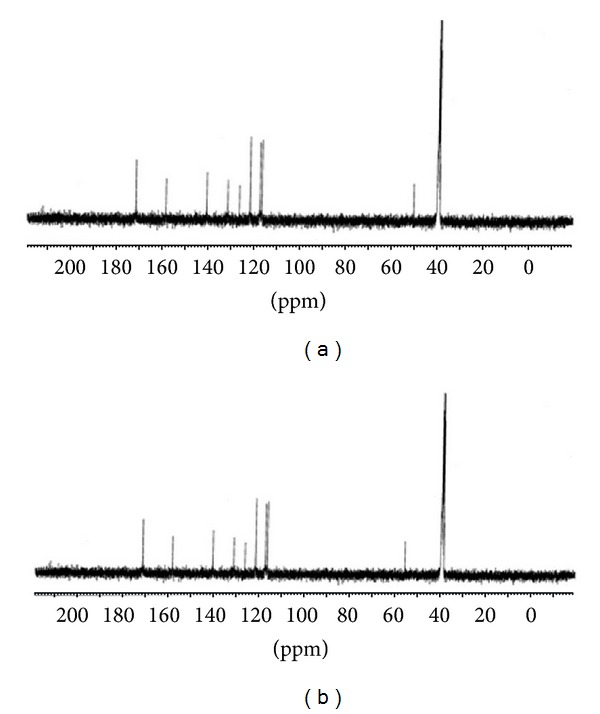
^13^C-NMR spectra of (a) BEF ligand and (b) BEF–Zn.

The ^13^C NMR spectra of the BEF terpolymer ligand and its Zn^2+^ metal complex are shown in Figure 5. The observed chemical shifts are assigned on the basis of the literature [22, 27]. The spectrum of ligand showed that the corresponding peaks at 171.7, 158.5, 131.5, 121.9, 140.6, 116.7, and 125.3 ppm are attributed to the aromatic carbons of the benzothiazole ring. The peak appearing at 50.2 ppm is assigned to the methylenic carbon of NH–CH_2_ bridge in the ligand. The spectrum of Zn^2+^ complex showed the peaks for aromatic ring in the same region which was appearing in its parent ligand. The shifting of the methylene carbon from 50.2 to 56.1 ppm from its ligand clearly suggests that the coordination takes place between the nitrogen atoms of the ethylenediamine moiety.

**Figure 6:**
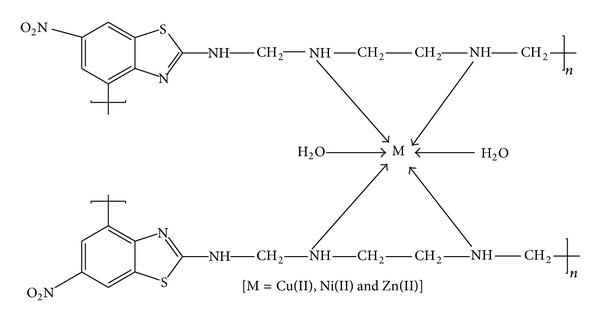
Proposed geometry of BEF metal complexes.

From the elemental and spectral data (FTIR, UV-Vis., ESR, and NMR (^1^H and ^13^C)), the structure of the BEF terpolymer ligand and its metal complexes has been confirmed. The proposed geometry of the BEF complexes is shown in Figure 6.

### 3.6. Surface Analysis

**Figure 7:**
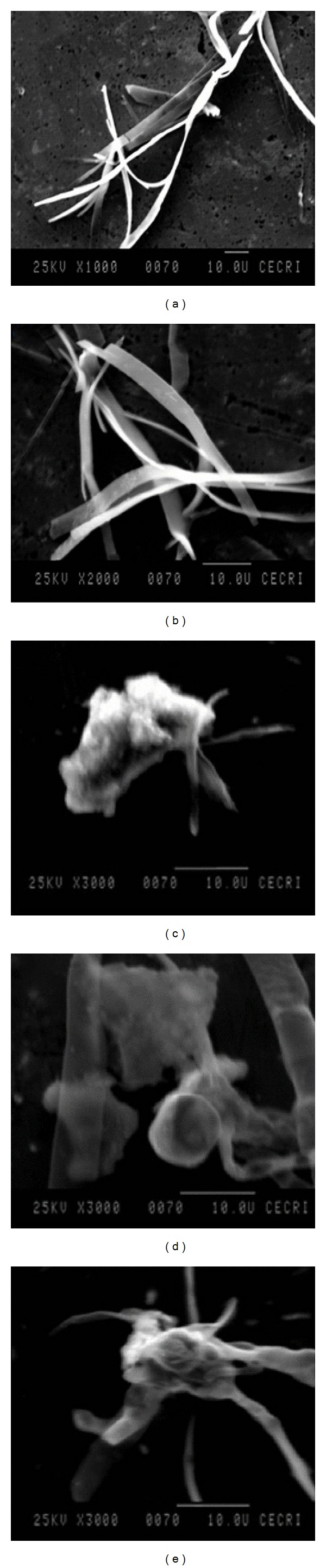
SEM images of (a) and (b) BEF ligand, (c) BEF–Cu, (d) BEF–Ni, and (e) BEF–Zn.

The SEM is applied to understand the surface features of the polymeric materials. The SEM photographs in ×1000 and ×2000 magnifications for BEF ligand and ×3000 magnifications for BEF complexes are shown in Figure 7. The white bar at the bottom of the SEM photographs represents the scale. The SEM photographs of the BEF terpolymer ligand show leaf like appearance. The scattered structure of the terpolymer establishes a transition state between the crystalline and the amorphous phases [28].

The morphology of BEF terpolymer resin exhibits growth of crystals from polymer solutions corresponding to the most prominent organization in polymers on a large scale with spherulites. Ideally, spherulites are the aggregates of submicroscopic size particles. Spherulites are characterized by secondary structural features, such as faint corrugations. The SEM photographs of polymers have an appreciable amorphous fraction with a small amount of shallow pits. The surface of BEF metal complexes contributes to greater segments of crystalline regions compared to their parent ligand which may arise from the contraction of the voids by the cooperative contribution of the ligand for complexation with metal ions or the disappearance of the voids in the rearrangement of polymer chains for complexation with metal ions. This is a further evidence for the complexation of BEF terpolymer with metal ions.

### 3.7. XRD Studies

**Figure 8:**
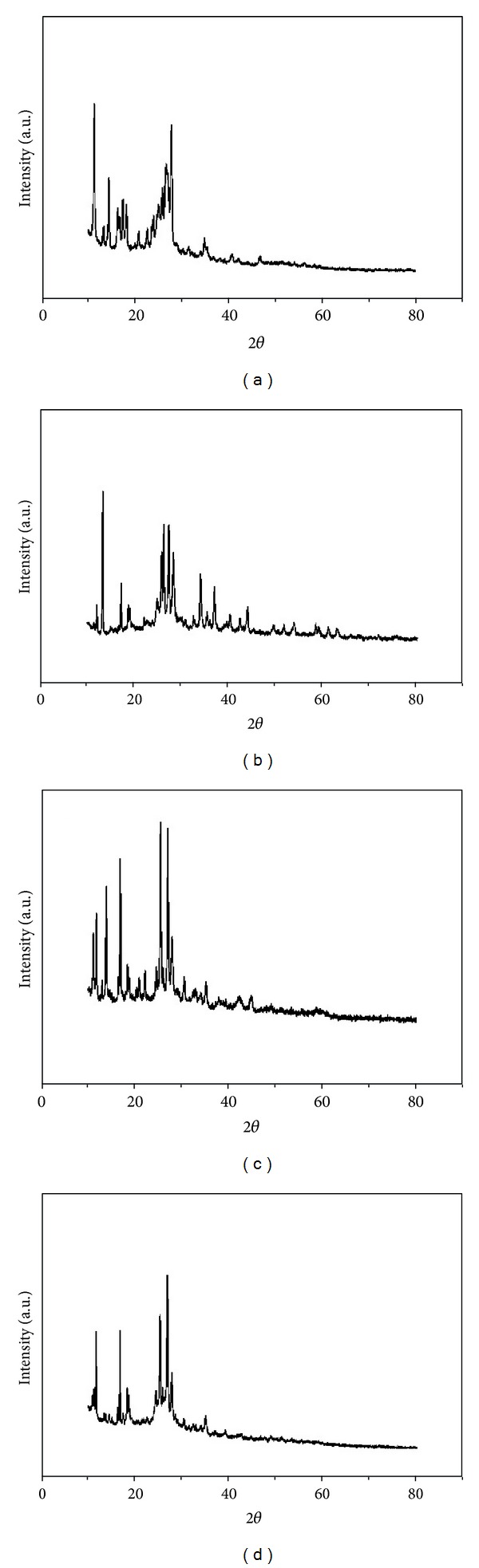
XRD patterns of (a) BEF ligand, (b) BEF–Cu, (c) BEF–Ni, and (d) BEF–Zn.

X-ray diffraction (XRD) patterns of BEF and its metal complexes are shown in Figure 8. It provides a detailed comparative account of the behavior of the terpolymer and its metal complexes. The pattern of BEF ligand shows the main characteristic peak and broad shoulder. In view of the relative half value width, it may be concluded that the polymer is partially crystalline and the broad characteristic peak indicates the amorphous nature of the terpolymer. Therefore, the BEF terpolymer has transition state between amorphous state and crystalline state, whereas its metal chelates possess crystalline nature. The enhanced crystalline behavior of the BEF complexes may be due to the insertion of metal ions with their parent ligand [29].

### 3.8. Thermal Stability

**Figure 9:**
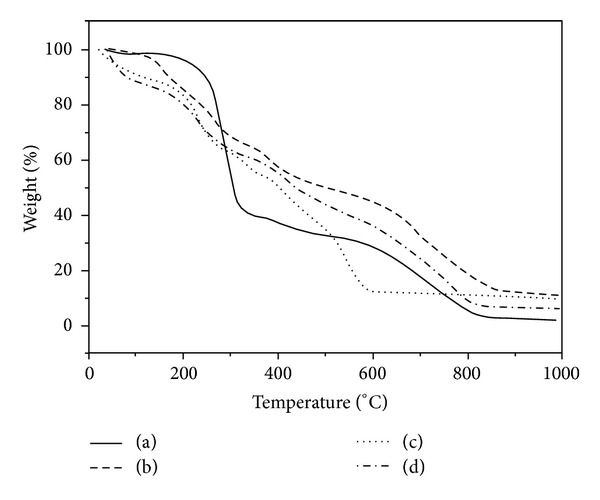
TGA of (a) BEF ligand, (b) BEF–Cu, (c) BEF–Ni, and (d) BEF–Zn.

Thermal stability of the BEF terpolymer and its metal complexes was analyzed by thermogravimetric analysis (TGA). The thermograms of the terpolymer and its metal complexes are shown in Figure 9 and their percentages of weight loss at various temperatures are presented in Table 4.

#### 3.8.1. Thermal Stability of BEF Terpolymer

From the thermogram of BEF terpolymer, there were three stages of degradation (200–320°C, 320–580°C, and 580–835°C) observed. The first stage of degradation was observed between 200°C and 320°C (found: 58.83%, calc.: 57.94%) which reveals the degradation of the benzothiazole ring. The second stage of degradation begins at 320°C and ends at 580°C (found: 10.03%, calc.: 09.31%) which may be due to the elimination of cross-linkages. The third and final stage of degradation is observed above 580°C and continued up to 825°C (found: 25.06%, calc.: 24.73%), which can be attributed to the weight loss of aliphatic group (ethylenediamine moiety) from the BEF terpolymer.

#### 3.8.2. Thermal Stability of BEF Metal Complexes

In general, the water of hydration may be considered as either crystal water molecules or coordination water molecules. According to Nikolaev et al. [30] water eliminated below 150°C can be considered as crystal or lattice water and water eliminated above 150°C may be due to its coordination to the metal atom present in chelates. In the present study, in the case of BEF–Cu^2+^, Ni^2+^, and Zn^2+^ complexes, the removal of water from the complex was completed within 150°C. These water molecules are probably crystal or lattice water molecules. The removal of water molecules observed in the range of 150–200°C (found: 5.59–6.08%, calc.: 5.45–5.51%) of all the metal chelates is due to the elimination of two coordinated water molecules. These observations further confirm the distorted octahedral geometry of BEF metal complexes.

The thermogravimetric curves of all the BEF metal chelates showed two degradation steps after loss of water molecules, which may indicate that the first step (200–600°C) is faster than the second step (600–860°C). This may be due to the fact that the noncoordinated part of the ligand decomposes first while the coordinated part decomposes later and finally it may form the representative metal oxides. The weight of the residues confirms fairly well that of metal oxides (found: 12.47–13.62%, calc.: 11.43–12.32%).

The thermal degradation data of the BEF terpolymer and its metal complexes reveal that the polymeric complexes show better stability than the BEF terpolymer due to the coordination of metal ions. The order of thermal stability of the BEF polymer and its complexes is BEF–Cu^2+^ > BEF–Zn^2+^ > BEF ligand > BEF–Ni^2+^. The greater stability of the metal complexes may be due to the coordination and the enhanced cross linking nature with crowd effect of the metal complexes [31]. In the case of Ni^2+^ complex, the degradation occurred exceptionally at low temperature compared to their ligand, which may be due to the oxidation of polymer by the catalytic action of the metal ion [32].

#### 3.8.3. Thermal Kinetics

**Figure 10:**
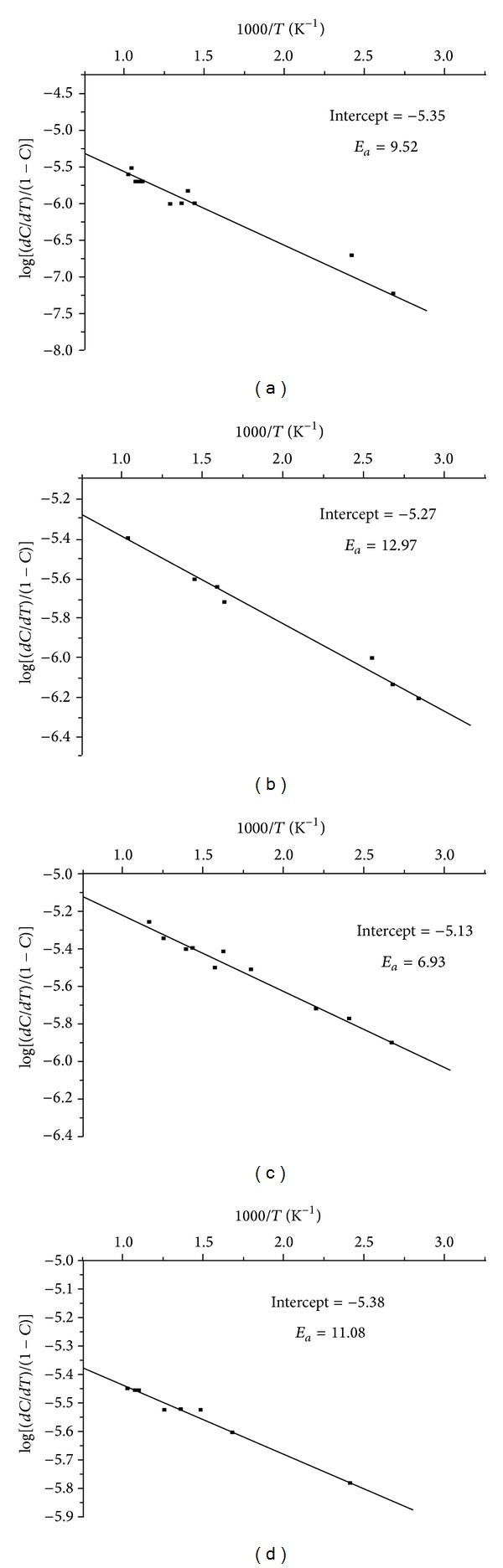
Sharp-Wentworth plots of (a) BEF ligand, (b) BEF–Cu, (c) BEF–Ni, and (d) BEF–Zn.

**Figure 11:**
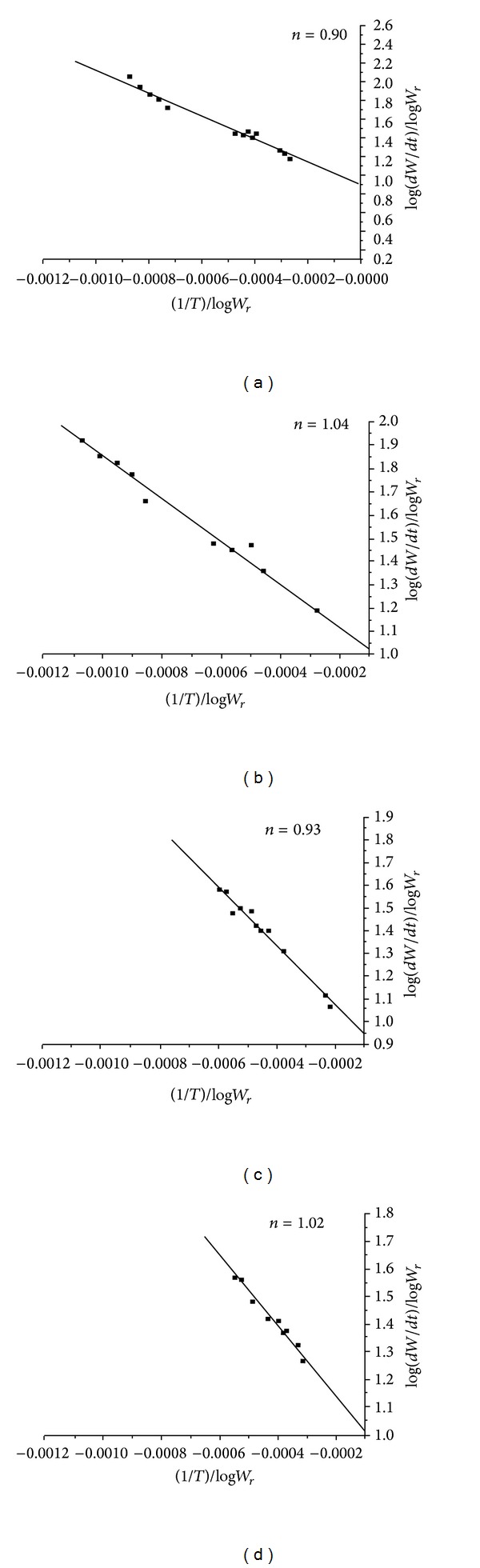
Freeman-Carroll plots (*n*) of (a) BEF ligand, (b) BEF–Cu, (c) BEF–Ni, and (d) BEF–Zn.

**Figure 12:**
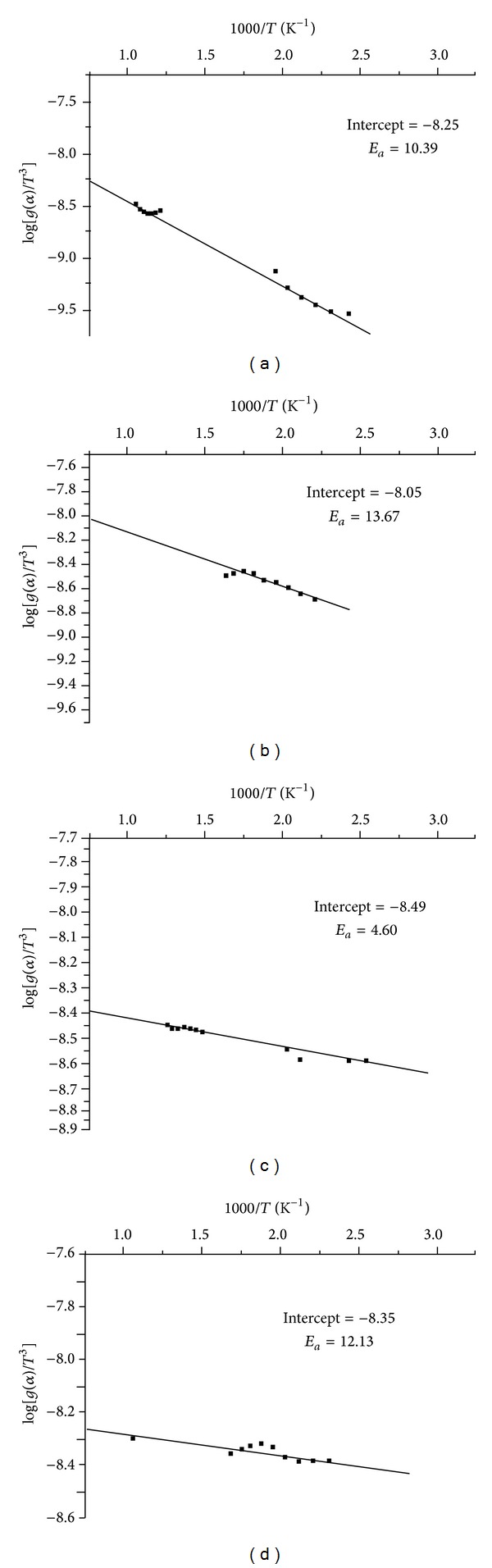
Freeman-Carroll plots (*E*_*a*_) of (a) BEF ligand, (b) BEF–Cu, (c) BEF–Ni, and (d) BEF–Zn.

(*1) Sharp-Wentworth and Freeman-Carroll Method.* Thermal kinetic analysis was performed using Sharp-Wentworth method and Freeman-Carroll methods. The activation energy (*E*_*a*_) and order of reaction (*n*) plots for BEF terpolymer ligand and its metal complexes by Sharp-Wentworth and Freeman-Carroll methods are shown in Figures 10, 11, and 12. The expressions used to calculate the kinetic and thermodynamic parameters are presented as follows.

(i) Entropy change (Δ*S*):
  (2) $$\begin{array}{c} \text{Intercept}=\log\frac{KR}{h\Phi E_{a}}+\frac{\Delta S}{2.303R}, \end{array}$$
 where *K* = 1.3806 × 10^−16^ erg/deg/mole, *R* = 1.987 cal/deg/mole (8.314 J/K/Mol), *h* = 6.625 × 10^−27^ erg sec, Φ = 0.166, Δ*S* = change  in  entropy, and *E*_*a*_ = activation  energy  from  graph.
(ii) Free energy change (Δ*F*):
  (3) $$\begin{array}{c} \Delta F=\Delta H-T\Delta S, \end{array}$$
 where Δ*H* = enthalpy change = activation energy, *T* = temperature in *K*, and Δ*S* = result from  (2).
(iii) Frequency factor (*Z*):
  (4)  B2/3=log⁡ZEaRΦ,
  (5)  B2/3=log⁡3+log⁡[1−31−α]−log⁡p(x),
 where *Z* is the frequency factor, *B* is calculated from (5), log⁡*p*(*x*) is calculated from Doyle's table corresponding to activation energy, and *α* is degree of transformation (*α* = *w*/*W*_*c*_).
(iv) Apparent entropy (S*):
  (6) $$\begin{array}{c} S^{*}=2.303\log\frac{Zh}{RT^{*}}, \end{array}$$
 where *Z* is calculated from (4) and T* is half decomposition temperature.

Further from the knowledge of activation energy calculated by Freeman-Carroll method, it is possible to evaluate the values of various thermodynamic parameters. The order of reaction (*n*) and activation energy (*E*_*a*_) and the thermodynamic parameters such as frequency factor (*Z*), entropy change (Δ*S*), free energy change (Δ*F*), and apparent entropy (S*) are presented in Table 5. The *E*_*a*_ values calculated by SW and FC methods are in good agreement with each other. The order of activation energies for BEF ligand and its Cu^2+^, Ni^2+^, and Zn^2+^ complexes is parallel to the order of their thermal stability [32]. The values of thermodynamic parameters were comparable indicating a common mode of decomposition reaction. The abnormal low values of frequency factor (*Z*) indicate that the decomposition reaction of BEF terpolymer and its metal complexes can be depicted as a slow reaction. The order of reactions (*n*) of BEF ligand and its metal complexes is found to be 0.90, 1.04, 0.93, and 1.02, respectively. Hence, the BEF terpolymer and its meal complexes may be following nearly a first order kinetics.

(*2) Phadnis-Deshpande Method.* The activation energies calculated for BEF ligand and its metal complexes using Phadnis-Deshpande method are presented in Table 6. From the *E*_*a*_ values, it is possible to propose the degradation reaction mechanism using PD method. Further the *E*_*a*_ values calculated by PD method are in good agreement with FC and SW methods. Based on the closest *E*_*a*_ values calculated by PD, SW, and FC methods, the Power law mechanism suits very well for the BEF ligand and BEF–Cu and BEF–Zn metal complexes. However, BEF–Ni complex follows the Brounshtein-Ginstling, 3-dimensional diffusion mechanism.

### 3.9. Biological Studies

#### 3.9.1. Antibacterial Activity

To determine the antibacterial activity of the BEF terpolymer and its Cu^2+^, Ni^2+^, and Zn^2+^ complexes, the disc diffusion method was used, with amoxicillin as the standard antibiotic. The prepared compounds were tested against* Shigella sonnei*,* Escherichia coli*,* Klebsiella *species*, Staphylococcus aureus*,* Bacillus subtilis*, and* Salmonella typhimurium* microorganisms.

Table 7 contains the screening results corresponding to the polymeric ligand and its metal complexes with fair inhibition of growth against the tested organisms.* Shigella sonnei* is a nonmotile, non-spore-forming, and facultative anaerobic gram-negative bacterium. Shigella bacteria multiply within colonic epithelial cells and cause mucosal ulceration, inflammation, bleeding, high fever, malaise, diarrhea, and tenesmus. The ligand and its metal complexes showed a reasonable activity against the* S. sonnei* species.* E. coli* is an aerobic, gram-negative, and rod shaped bacterium. Infection of* E. coli* can lead to bloody diarrhea and kidney failure. The polymeric ligand and its complexes hold a modest activity against the* E. coli*.* Klebsiella* is a genus of nonmotile and gram-negative bacteria that infects sites such as nasal passages, liver, lung, urinary tract, bloodstream, eye, bone, and joints. It causes a wide range of disease states, notably pneumonia, septicemia, soft tissue infections, biliary tract infection, infection of the upper and lower respiratory tract, and liver abscess. The BEF ligand and its metal complexes showed a reasonable result against the growth of* Klebsiella*.* Staphylococcus aureus*, a gram-positive and spherical bacterium, leads to life-threatening diseases like pneumonia, osteomyelitis, endocarditis, and toxic shock syndrome. Toxic shock syndrome is characterized by the sudden onset of high fever, vomiting, diarrhea, and muscle aches, followed by low blood pressure (hypotension), which can lead to shock and death. There may be a rash resembling sunburn with peeling of skin. The ligand and its complexes showed a humble activity against the growth of* S. aureus*.* Bacillus subtilis* are rod-shaped and nonpathogenic gram-positive bacteria. The BEF ligand and complexes showed superb activity against the growth of this bacterium.* Salmonella typhimurium* is a pathogenic gram-negative bacteria predominately found in the intestinal lumen. When the bacterial cells enter epithelial cells lining the intestine they cause host cell ruffling which temporarily damages the microvilli on the surface of the cell. This causes a rush of white blood cells into the mucosa and leads to diarrhea. The ligand and its metal complexes showed a moderate activity against the growth of the* typhi* species.

A comparative account on the effect of antibacterial activity against the chosen microbes indicates that the ligand and its three metal chelates show reasonable activity against* Shigella sonnei* (15, 21, 18, and 16 mm) and* Bacillus subtilis* (15, 18, 20, and 17 mm) while the growth of other organisms is moderately inhibited by the BEF ligand and its Cu^2+^, Ni^2+^, and Zn^2+^ chelates. The highest antibacterial action (21 mm) has been observed for BEF–Cu^2+^ complex against* Shigella sonnei*.

#### 3.9.2. Antifungal Activity

The antifungal activity of the BEF ligand and its metal complexes has also given interesting results and the growth inhibitions against the chosen fungal strains are tabulated in Table 8. All the complexes and their ligand have an excellent activity against* Aspergillus flavus*, a mold type fungal strain which may invade arteries of the lung or brain to cause infections and also produce a toxin.* Aspergillus niger* is one of the most common causes for otomycosis. The chelates and the ligand are found to have a reasonable activity for* A. niger*.* Penicillium* species causes infection in humans and the resulting disease is known generically as penicilliosis. The ligand and the metal complexes have wonderful activity against this species. Further, BEF–Cu complex has a superior activity compared to the rest of other complexes.* Candida albicans* is a diploid fungus and a causal agent of opportunistic oral and genital infections in humans and also emerged as an important cause of morbidity and mortality in immunocompromised patients. The BEF ligand and its chelates possess a modest activity against* C. albicans* over and above the standard.* Cryptococcus neoformans* is an encapsulated yeast-like fungus that can live in both plants and animals and cause lung infection. The terpolymer ligand and its chelates have a fair activity against this fungal strain. The chelate compounds and the ligand have moderate activity against* Mucor *species, a filamentous fungus which causes septic arthritis, renal infections, and pulmonary infections.

From the antimicrobial results, the metal complexes show higher activity than their ligand which may be strongly dependent on the central metal ions and coordination numbers metal chelates. The higher activity due to the metal ions shared with the donor atoms (N and S) of the thiazole ring is present in the ligand and the *π*-electron delocalization over the chelate ring. This effect increases the lipophilic character of the metal ion, which favors the permeation through the lipoid layers of the bacterial and fungal membranes [33, 34]. It is perceived that the factors such as solubility, conductivity, dipole moment, and cell permeability may also contribute to the increased activity of the complexes [35, 36]. From the results of the studies, at 500 ppm concentration, the compounds establish better antimicrobial activity. Hence it is an interesting result that a very low concentration of the compound, namely, 500 ppm, is enough to bring out an effective inhibition against the chosen microbes.

## 4. Conclusion

The synthesized 2-amino-6-nitro-benzothiazole-ethylenediamine-formaldehyde terpolymer has acted as a ligand successfully to form metal complexes. The synthesized compounds were well characterized by various spectral and physical analyses. The morphological features suggest that the complexes are slightly more crystalline in nature than their parent ligand. The antibacterial and antifungal activities reveal that the terpolymer and its metal complexes can be active against the selected bacterial and fungal strains. Nearly all of the compounds may suit well for drug delivery field after checking for their toxicology behaviour in thorough manner. Thermal studies are also helpful to realize the standing capacity of the compounds which survive as antimicrobial coating materials in various environmental conditions. Because of the proven high stability nature and microbial prevention capacity, the BEF terpolymer and its Cu^2+^, Ni^2+^, and Zn^2+^ complexes hold inadequate applications in biological field. The thermal stability of the terpolymer and its metal complexes was found greater. From the thermodynamic and kinetic studies, the abnormal low values of frequency factor (*Z*) indicate that the thermal decomposition reaction of BEF terpolymer and its metal complexes can be depicted as a slow reaction. The BEF terpolymer and its metal complexes may be following nearly a first order kinetics.

## Figures and Tables

**Table 1:** Physicochemical and analytical data.

Compound	Empirical formula of the repeating unit	Formula mass	Elemental analysis (%)					Λ (Ω^−1^ cm^2^ mol^−1^)
			Found (calc.)					
			C	H	N	S	M	
BEF ligand	C_11_H_13_N_5_O_2_S	279.36	47.09 (47.29)	4.16 (4.35)	25.21 (25.49)	11.20 (11.39)	—	—
BEF–Cu	C_22_H_26_N_10_O_4_S_2_Cu*·*2H_2_O	658.23	40.71 (40.92)	4.39 (4.62)	21.74 (21.94)	9.19 (9.27)	9.31 (9.62)	132.05
BEF–Ni	C_22_H_26_N_10_O_4_S_2_Ni*·*2H_2_O	653.34	40.25 (40.37)	4.47 (4.79)	21.29 (21.46)	9.62 (9.83)	9.26 (9.45)	98.37
BEF–Zn	C_22_H_26_N_10_O_4_S_2_Zn*·*2H_2_O	660.10	40.23 (40.52)	4.23 (4.45)	21.38 (21.53)	9.12 (9.29)	9.41 (9.70)	113.29

C: carbon, H: hydrogen, N: nitrogen, S: sulphur, and M: metal ion.

**Table 2:** FTIR data.

Compound	Observed frequencies (cm^−1^)			
	BEF ligand	BEF–Cu	BEF–Ni	BEF–Zn
*ν* NH (asym. and sym.)	3455−3297	3408–3296	3348–3295	3345–3280
*ν* NH (bending)	1532	1527	1526	1521
CH (stretching)	2731	2697	2712	2725
*ν* CH_2 _(asym. and sym.)	3078–2936	3093–2912	3098–2932	3087–2945
*ν* CH_2_ (bending in N–CH_2_–CH_2_–N)	1450	1448	1449	1446
2,6,8-tri substitution (benzothiazole)	1296−825	1298–825	1293–827	1292–825
*ν* N→M	—	432	433	433
*ν* C–NO_2_	1505–1518	1503–1576	1524–1575	1513–1583
*ν* C=N	1540	1590	1586	1580
*ν* C–S–C	740	753	763	748

**Table 3:** UV-Visible, ESR data, and magnetic moments.

Compound	Transitions (cm^−1^)	Assignments	Geometry	ESR		Magnetic moment (B.M)
				g_II_	g_⊥_	
BEF	260 nm	*π* → *π*∗	—	—	—	—
	370 nm	*n* → *π*∗				

BEF–Cu	14295	^1^ *B*_*g*_ → ^2^ *E*_*g*_	Octahedral	2.21	2.11	1.85
	16465	^1^ *B*_*g*_ → ^2^ *E*_*g*_				
	24580	Charge transfer				

BEF–Ni	12474	^3^ *T*_2*g*_(*F*)*←* ^3^ *A*_2*g*_(*F*),	Octahedral	—	—	3.05
	14642	^3^ *T*_1*g*_(*F*)*←* ^3^ *A*_2*g*_(*F*)				
	24370	^3^ *T*_1*g*_(*P*)*←* ^3^ *A*_2*g*_(*F*)				

BEF–Zn	—	—	Octahedral	—	—	Diamagnetic

**Table 4:** TGA data for thermal stability.

Compound	Percentage of weight loss at various temperatures								*T*_50_^∗ ^ (°C)	*T*_max⁡_^∗∗ ^ (°C)
	100	200	300	400	500	600	700	800 (°C)		
BEF ligand	01.53	03.65	44.14	62.36	67.18	71.40	82.24	94.42	310	825
BEF−Cu	01.94	14.32	31.80	43.01	49.96	55.49	66.86	81.22	500	860
BEF−Ni	08.93	16.60	37.35	49.35	64.60	87.67	—	—	405	650
BEF−Zn	14.64	22.62	38.33	46.16	57.26	64.92	76.14	91.29	425	830

*Temperature of 50% weight loss; ∗∗maximum decomposition temperature.

**Table 5:** Kinetic and thermodynamic parameters data.

Compound	Activation energy (*E*_*a*_/kJ mol^−1^)		Entropy change (Δ*S*/J)	Free energy change (Δ*F*/kJ)	Frequency factor (*Z*/s^−1^)	Apparent entropy (S*/J)	Order of reaction (*n*)
	∗SW	∗∗FC					
BEF ligand	9.52	10.39	−158.11	59.40	1310	−22.95	0.90
BEF−Cu	12.97	13.67	−154.25	90.79	1205	−23.31	1.04
BEF−Ni	6.93	4.60	−162.90	70.57	1537	−22.94	0.93
BEF−Zn	11.08	12.13	−160.01	80.13	1243	−23.18	1.02

*SW: Sharp-Wentworth; ∗∗FC: Freeman-Carroll.

**Table 6:** Activation energies calculated by Phadnis-Deshpande method.

Reaction mechanism model	Energy of activation (*E*_*a*_) (kJ/mol)			
	BEF ligand	BEF−Cu	BEF−Ni	BEF−Zn
Power law	6.0932	6.2642	5.9691	5.4312
Power law	**12.1864**	**12.5284**	11.9382	**10.8625**
Phase boundary (contracting sphere)	1.6497	1.6816	1.6174	1.5009
Phase boundary (contracting cylinder)	2.5894	2.6436	2.5383	2.3466
Nucleation and nuclei growth (Avrami-Erofeev nuclei growth)	1.2135	1.3830	1.1756	0.7447
Nucleation and nuclei growth (Avrami-Erofeev nuclei growth)	0.8090	0.9220	0.7837	0.4964
Nucleation and nuclei growth (Avrami-Erofeev nuclei growth)	0.6067	0.6915	0.5878	0.3723
Valensi, 2-dimensional diffusion	16.8354	17.1926	16.5034	15.2695
Jander, 3-dimensional diffusion	3.2995	3.3632	3.2349	3.0018
Brounshtein-Ginstling, 3-dimensional diffusion	5.6520	5.5015	**5.5669**	5.7981

The values indicated in bold are closest to the *E*_*a*_ value estimated by FC and SW methods.

**Table 7:** Antibacterial activity of BEF ligand and its complexes.

Compound	Inhibition zone (mm)					
	*S. sonnei *	*E. coli *	*Klebsiella *sp.	*S. aureus *	*B. subtilis *	*S. typhi *
BEF ligand	15	13	13	14	15	12
BEF–Cu	21	17	17	18	18	18
BEF–Ni	18	15	18	17	20	15
BEF–Zn	16	15	15	16	17	15
Standard	26	28	26	25	27	28
Control	—	—	—	—	—	—

Standard: amoxicillin; control: DMSO.

**Table 8:** Antifungal activity of BEF ligand and its complexes.

Compound	Inhibition zone (mm)					
	*Aspergillus flavus *	*Aspergillus niger *	*Penicillium *species	*Candida albicans *	*Cryptococcus neoformans *	*Mucor*species
BEF ligand	14	12	12	13	11	12
BEF–Cu	20	18	19	18	15	19
BEF–Ni	17	14	17	15	13	13
BEF–Zn	15	15	18	17	14	16
Standard	20	21	19	21	18	22
Control	—	—	—	—	—	—

Standard: fluconazole; control: DMSO.
